# Highly Stable Photoluminescent CeF_3_ Nanocrystal as a Versatile Probe for Neurotoxic Alkaloid (Anabasine) Sensing via Fluorescence Modulation

**DOI:** 10.1021/acs.analchem.5c05171

**Published:** 2026-02-09

**Authors:** Deepak Dabur, Jie Li, Priyanka Rana, Hui-Fen Wu

**Affiliations:** † Department of Chemistry, National Sun Yat-Sen University, Kaohsiung, 70, Lien-Hai Road, Kaohsiung 80424, Taiwan; ‡ School of Pharmacy, College of Pharmacy, Kaohsiung Medical University, Kaohsiung 807, Taiwan; § Institute of Medical Science and Technology, National Sun Yat-Sen University, Kaohsiung 80424, Taiwan; ∥ School of Medicine, College of Medicine, National Sun Yat-Sen University, Kaohsiung 80424, Taiwan; ⊥ Institute of Precision Medicine, National Sun Yat-Sen University, Kaohsiung 80424, Taiwan; # Institute of Biopharmaceutical Science, National Sun Yat-Sen University, Kaohsiung 80424, Taiwan

## Abstract

This study introduces highly stable photoluminescent CeF_3_ nanocrystals as a first ratiometric fluorescent probe for the selective detection of the neurotoxic alkaloid anabasine in environmental water matrices. The hexagonal-phase CeF_3_ nanocrystals (40–50 nm) exhibit dual-emission bands at 322 (quenched) and 433 nm (enhanced) upon anabasine binding, enabling sensitive (LOD: 0.17 μM) and matrix-resistant quantification. Structural (XRD, TEM, EDS) and optical (pH and thermal stability: pH 4–10, 30–90 °C) characterizations confirm robustness for real-world applications. Recovery assays (95–103%) in lake, river, and tap water demonstrate minimal matrix interference, outperforming LC-MS/MS and GC-FID methods that require derivatization or complex sample pretreatment. The cost-effectiveness, simplicity, and ratiometric self-calibration of the method highlight its potential for on-site environmental monitoring of tobacco-derived contaminants.

## Graphical abstract

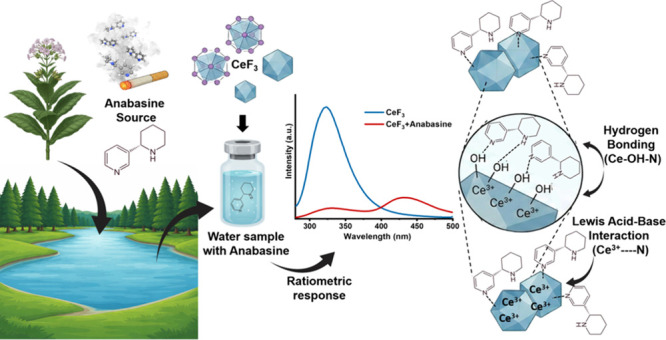

## Introduction

Inorganic fluorides, particularly those incorporating rare earth metals, have been widely used recently due to their large band gaps, high refractive index, low toxicity, thermal stability, and low nonradiative transition scattering rate.^1–4^ Among these, cerium being the most abundant rare earth metal stands out and exhibits two different oxidation states, +3 and +4. This study is focused on the optically active trivalent state, which generates luminescence in UV and VUV regions.^5^ Unlike most rare earth ions, Ce^3+^ features parity-allowed 5d → 4f electronic transitions, which are sensitive to the environment because of the nonexistence of outer electron shielding.^6^ Cerium fluorides are being used as scintillating materials, catalysts,^7^ for photodynamic therapies,^8^ security inks,^9^ and as lasers^10^ and solid lubricants.^11^ As per reports, in biomedicine cerium fluoride (CeF_3_) is the best scintillator material.^12^ Additionally, the luminescence behavior of CeF_3_ can be finely tuned by modifying the type of crystal lattice with temperature, enhancing its potential for sensing technologies.^3,13^ The luminescence of Ce^3+^ arises from 4f → 5d transitions, which are highly sensitive to the ligand field, making the surrounding anion environment (e.g., O^2–^, F^–^, Cl^–^) critical for tuning emission properties.^14^ CeF_3_ is an attractive candidate for biomedical applications due to its low solubility and extremely low toxicity. Unlike CeO_2_, CeF_3_ contains Ce^3+^ as the sole oxidation state, providing a model system to study the redox-independent effects of cerium in biological environments.

While nicotine is the main tobacco alkaloid, minor alkaloids like anabasine and anatabine are increasingly used as biomarkers to distinguish tobacco users from nicotine replacement therapy (NRT) users.^15,16^ These compounds can act as the primary indicators of active smoking, as they are present in tobacco but not in pharmaceutical-grade nicotine.^17^ Anabasine, excreted from smokers into rivers, lakes, and wastewater, serves as a reliable biomarker for estimating smoking populations, avoiding overestimation caused by nicotine from replacement therapies.^18–20^

There have been plenty of studies based on anabasine detection. As reported, tobacco exposure can be distinguished with high specificity with quantification limits as low as 2–3 ng/mL in urine samples.^21^ However, these studies were applied with mass spectrometry (MS). Although a lower detection limit can be achieved with MS, the high cost and long sample pretreatment process are difficulties that are encountered. Furthermore, the traditional techniques like gas/liquid chromatography coupled with mass spectrometry pose challenges like isobaric interference.^22^ Therefore, fast, reliable, and cost-effective methods like fluorescence spectroscopy, electrochemical sensors, and single drop microextraction techniques have been recently used for the detection process. Additionally, anabasine not only acts as a biomarker but also shows pharmacological activity like acting on α7-nicotinic acetylcholine receptors, showing therapeutic effects in neurological disorders.^23^ This study aims to further investigate the detection and quantification of anabasine using fluorescent CeF_3_ nanocrystals (Scheme 1).

**Scheme 1:**
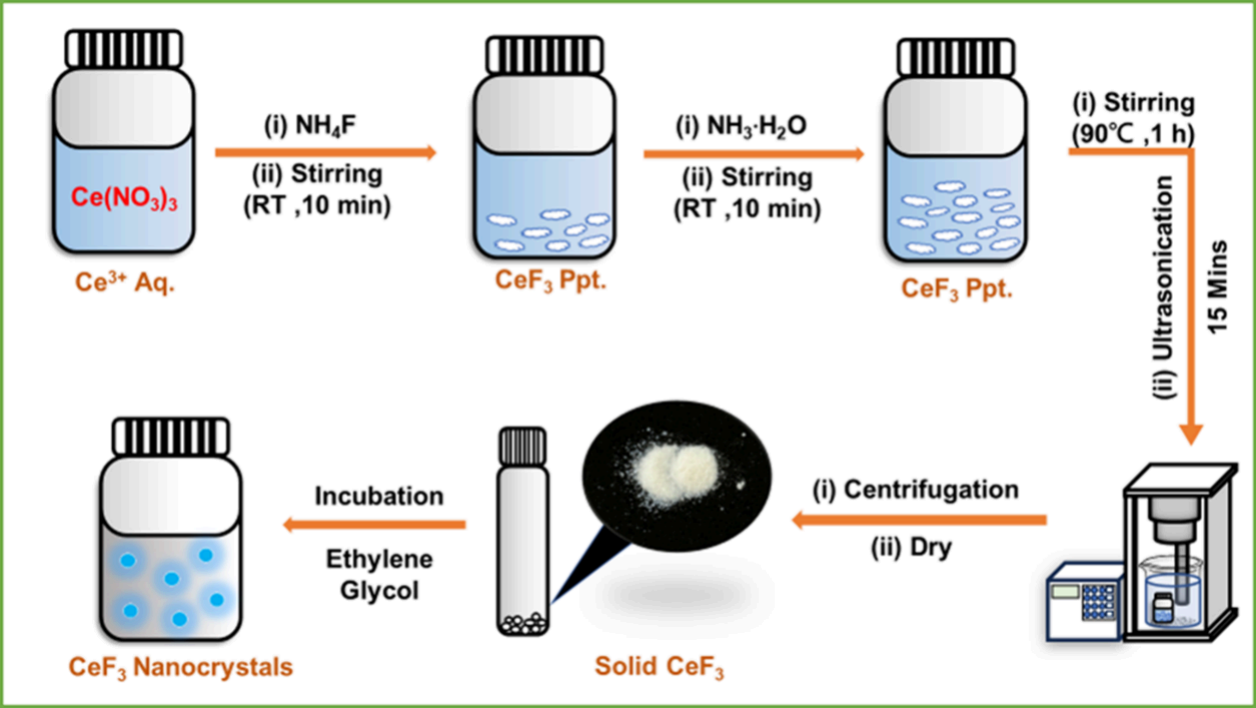
Synthesis of Fluorescent CeF_3_ Nanocrystals

## Results and Discussion

### Structure and Morphology Control of the Nanocrystal

The XRD pattern of the as-synthesized CeF_3_ (Figure 1a) shows sharp, well-defined peaks corresponding to a pure hexagonal phase (JCPDS No. 08-0045), confirming high crystallinity. Peaks at 2θ = 24.37°, 24.95°, 27.76°, 35.16°, 43.91°, 45.16°, 50.88°, 52.88°, 64.87°, 68.76°, and 71.19° were indexed to the (002), (110), (111), (112), (300), (113), (302), (221), (214), (304), and (411) planes, consistent with reported CeF_3_ data and validating the formation of the desired crystalline structure. Additional confirmation of CeF_3_ formation was obtained by FTIR spectroscopy (Figure 1b). The FTIR spectrum of CeF_3_ nanocrystals shows key absorption bands, confirming their formation. The peaks at 3220 (O–H stretch) and 1630 cm^–1^ (H–O–H bend) indicate surface-adsorbed water. The 1325 cm^–1^ band corresponds to Ce–F stretching vibrations, while the 720 cm^–1^ peak represents the IR-active lattice modes of CeF_3_, confirming its crystalline structure. These findings, along with the XRD data, validate the successful synthesis of CeF_3_ nanocrystals.

**Figure 1:**
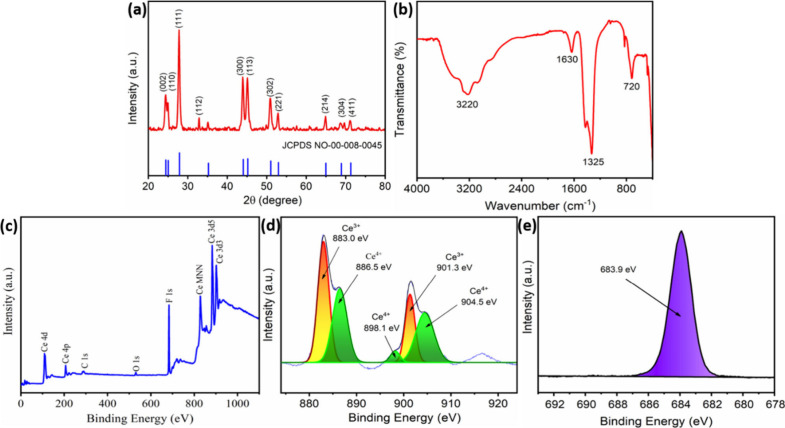
Structural characterization of CeF_3_ nanocrystals. (a) XRD pattern, (b) FTIR spectrum, and (c) XPS survey scan, (d) Ce 3d spectrum, and (e) F 1s spectrum.

The surface chemical composition and oxidation states of the CeF_3_ nanocrystals were investigated by using X-ray photoelectron spectroscopy (XPS) (Figure 1c). The Ce 3d spectrum (Figure 1d) reveals a mixed valence state of Ce^3+^ and Ce^4+^, with the characteristic peaks at 883.0 and 901.3 eV corresponding to Ce^3+^, while the peaks at 886.5, 898.1, and 904.5 eV are attributed to Ce^4+^.^24,25^ This indicates partial surface oxidation of Ce^3+^ to Ce^4+^. Additionally, the F 1s spectrum (Figure 1e) exhibits a strong peak at 683.9 eV, confirming the presence of fluorine in the CeF_3_ structure.^26^ These results demonstrate the coexistence of both oxidation states, which may influence the catalytic and optical properties of the material.

TEM analysis shows that the CeF_3_ nanocrystals have a narrow size distribution (40–50 nm) and high crystallinity, with well-defined lattice fringes (Figure 2a). The SAED pattern (Figure 2b) confirms the pure hexagonal phase (JCPDS No. 08-0045), and EDS analysis (Figure 2c) gives a Ce:F ratio of 64.8:35.2, matching the stoichiometric 1:3 ratio. Homogeneous elemental distribution and isotropic particle growth further verify the formation of phase-pure stoichiometric CeF_3_ nanocrystals. Figure S1 shows the volume- and number-based dynamic light scattering (DLS) size distributions of CeF_3_ nanocrystals, with Gaussian fitting and a low PDI of 0.22, confirming the narrow size dispersion and good colloidal uniformity. The photoluminescence (PL) properties and energy transfer mechanisms in CeF_3_ nanocrystals were systematically investigated to assess their potential for practical applications. PL spectra under varying excitation wavelengths revealed an optimal emission intensity at 260 nm excitation, confirming efficient fluorescence behavior (Figure 3a). To evaluate environmental stability, we examined the optical performance of the nanocrystals across different pH and temperature conditions—critical parameters for biological and chemical sensing applications. UV–vis and PL studies under these variable conditions are essential, as they (1) determine the robustness of the material in physio-environmental systems, (2) identify optimal working conditions for sensor applications, and (3) establish baseline stability for detecting analytes like anabasine or nicotine through fluorescence quenching mechanisms. These comprehensive optical characterizations provide fundamental insights into the potential of the nanocrystals as reliable fluorescent probes in complex matrices.

**Figure 2:**
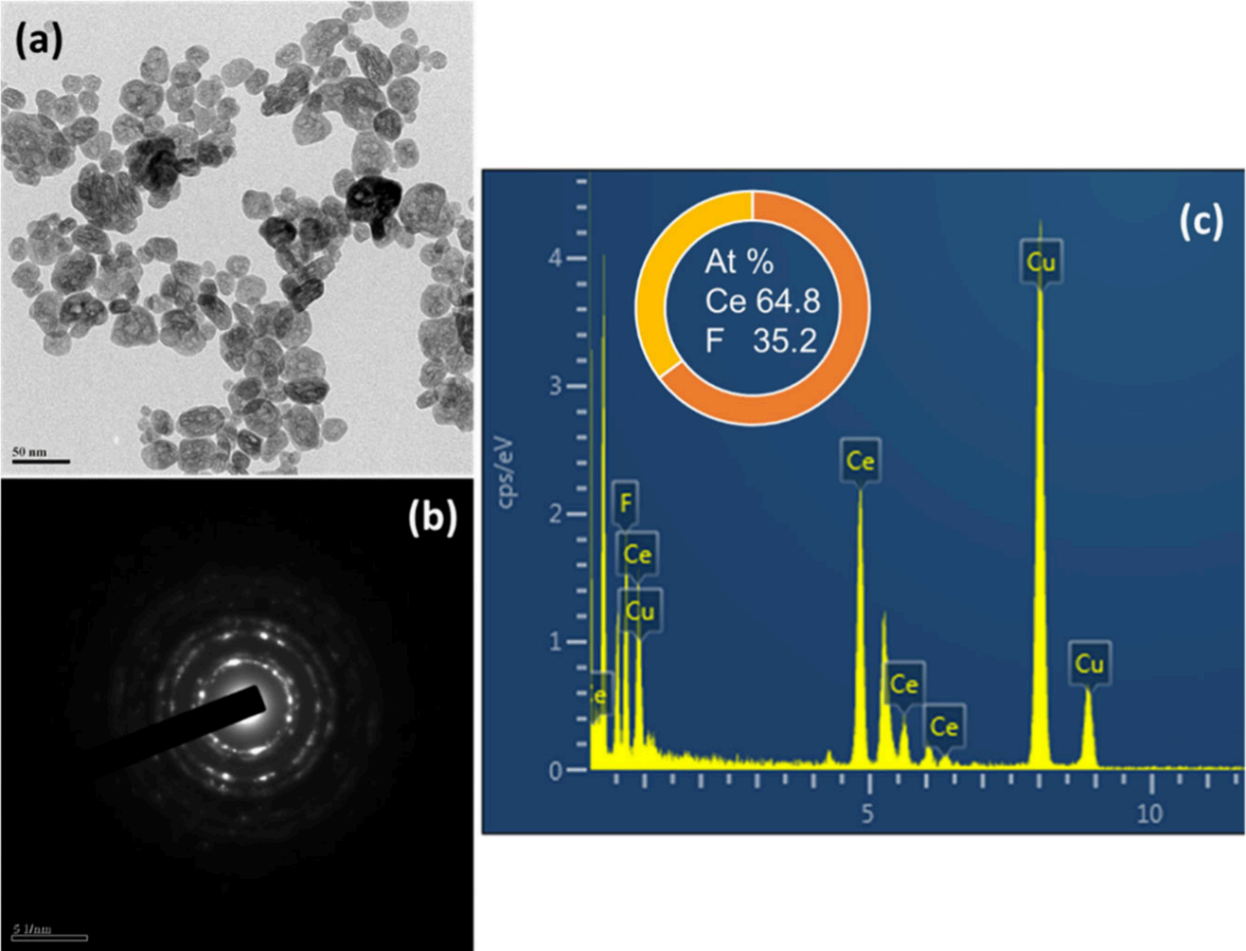
(a) TEM image, (b) SAED crystal pattern, and (c) EDS elemental spectrum for CeF_3_ nanocrystals.

**Figure 3:**
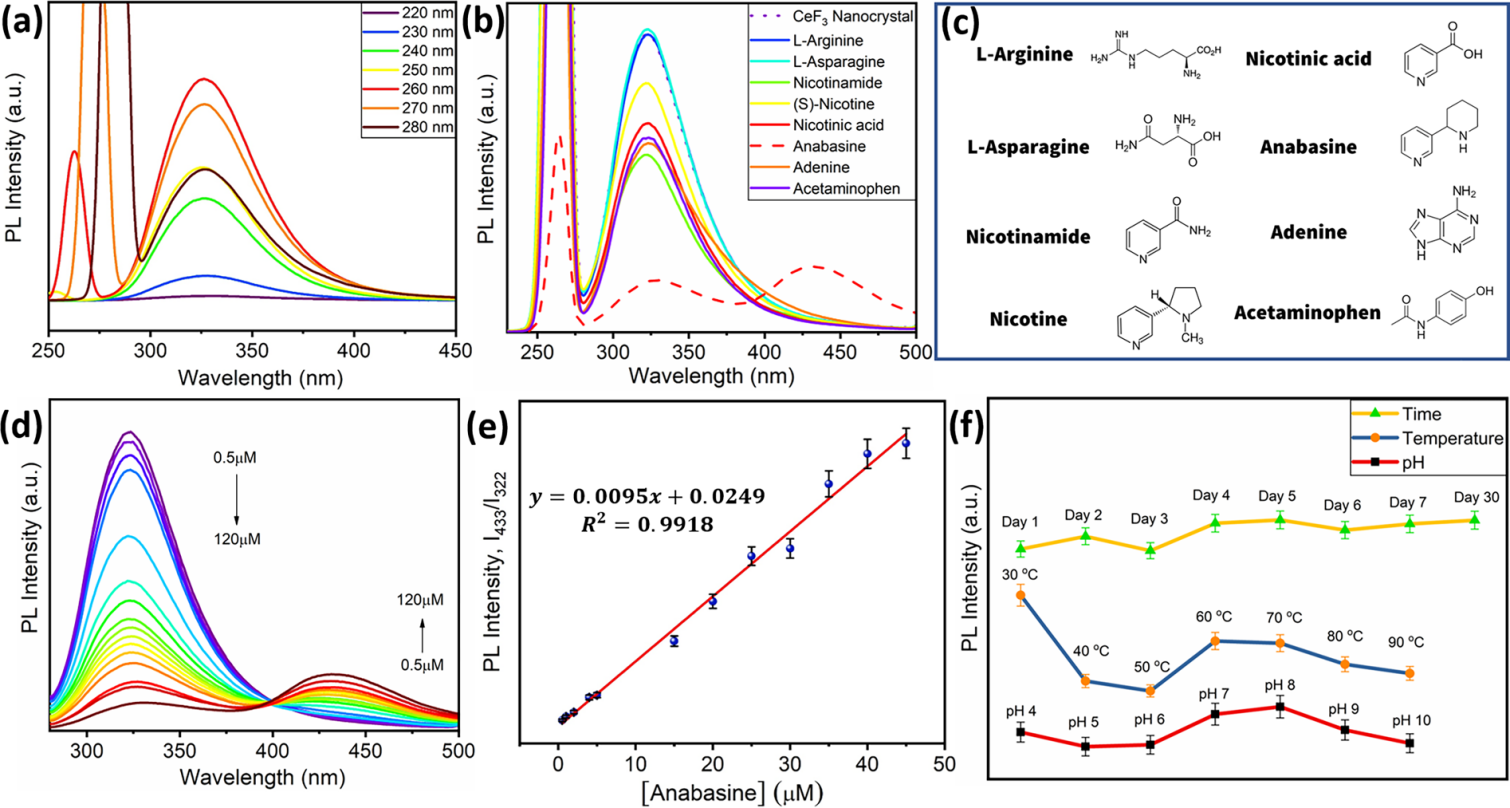
Optical properties and anabasine assay (*n* = 3). (a) PL data at different excitations, (b) selectivity study for different analytes, (c) structures of all analytes used in this study, (d) fluorescence response of CeF_3_ at varying anabasine concentrations, (e) calibration curve of ratiometric PL intensity (*I*_433_/*I*_322_) vs anabasine concentration (μM), and (f) stability data of CeF_3_ nanocrystals.

The binding interaction between CeF_3_ nanocrystals and the investigated alkaloids was characterized by significant optical changes, including measurable shifts in fluorescence intensity and emission wavelength, as well as observable naked-eye fluorescence variations. Spectrofluorimetric titrations were conducted by incrementally adding stock solutions of alkaloids (structures shown in Figure 3c) and nitrogen-blocked analogues (Figure S2) to a 10 mM ethylene glycol solution of CeF_3_ nanocrystals (Figure 3b). Notably, the emission spectra of anabasine exhibited distinct alterations, with the emergence of a new, highly intense band at 433 nm alongside the quenching of the original peak at 322 nm. This dual-peak response enables the ratiometric detection of anabasine, enhancing selectivity and sensitivity. In contrast, the fluorescence emission wavelength for all other alkaloid adducts remained constant at 322 nm, irrespective of the excitation wavelength, suggesting a unique binding mechanism specific to anabasine. The anabasine–CeF_3_ complex demonstrates an excitation-independent emission at 433 nm, indicative of a stabilized excited-state configuration distinct from other alkaloid interactions.

As illustrated in Figure 3d,e, the relative fluorescence changes of CeF_3_ nanocrystals upon titration with increasing concentrations of anabasine (in the micromolar range) demonstrate a clear selectivity over other tested alkaloids. Upon the incremental addition of anabasine, the intrinsic fluorescence of CeF_3_ at 322 nm progressively decreased, while a concomitant increase in emission intensity was observed at 433 nm, indicative of the formation of a distinct anabasine–CeF_3_ complex. The ratiometric response, quantified as the fluorescence intensity ratio *F*_433nm_/*F*_322nm_, exhibited a well-defined linear correlation (*R* ^2^ > 0.9917) within the anabasine concentration range of 0–45 μM, reaching saturation at the upper limit. This ratiometric approach minimizes environmental interference and enhances detection reliability. Based on the signal-to-noise (*S*/*N* = 3) criterion, the limit of detection (LOD) was determined to be 0.17 μM (eq S1), underscoring the sensitivity of the system for anabasine quantification. These results confirm that the CeF_3_ nanocrystals can serve as an effective fluorescent probe for selective, ratiometric anabasine detection, with potential applications in analytical and environmental monitoring.

Anabasine, a toxic alkaloid found in certain plants and pesticides, poses environmental and health risks due to its potential leaching into water systems from agricultural runoff or industrial discharge. Chronic exposure to anabasine-contaminated water can lead to neurological and respiratory toxicities in humans and aquatic life. Detecting anabasine in real-world samples such as river, pond, and tap water is critical for environmental monitoring, pollution control, and public health protection. According to wastewater-based epidemiology (WBE) studies conducted by environmental monitoring agencies and academic consortia in Europe and Australia, typical anabasine concentrations range from ∼9 to 38 ng/L.^27–29^ The CeF_3_ nanocrystal-based fluorescent sensor demonstrates exceptional suitability for real-world applications, owing to its pH stability (4–10) and thermal resilience over days as shown in Figure 3f, ensuring consistent performance across diverse aqueous environments. This stability is critical because anabasine detection relies on adsorption-induced surface complexation and ratiometric modulation, rather than sensor degradation. The ratiometric response (quenching at 322 nm and enhancement at 433 nm) provides intrinsic self-calibration, mitigating matrix interference and enhancing detection reliability in complex samples. Variations in recovery rates reflect water matrix effects on the sensor performance. Anabasine addition results in a significant quenching of CeF_3_ nanocrystal fluorescence with minimal interference from the investigated pesticides, pharmaceuticals, inorganic ions, and humic acid (Figure S3), highlighting the necessity of matrix-matched calibration for accurate quantification. River water particulates and dissolved metals cause modest fluorescence attenuation, whereas pond water organic matter may artificially elevate the apparent anabasine signal.

The mean recoveries of the target analyte across the three evaluated water bodies ranged from 95% to 103% (Table 1), demonstrating excellent analytical accuracy. Recovery values within this range indicate minimal matrix interference, as they closely align with the ideal 100% recovery benchmark for spiked samples. Statistical analysis confirmed no significant difference (*p* > 0.05) in recoveries between different spiking levels or water matrices, suggesting robust method performance independent of sample composition. The consistency in recoveries across varying conditions underscores the suitability of the method for environmental profiling, as it effectively mitigates matrix effects that could otherwise compromise data integrity.

**Table 1:** Recovery Table for Anabasine in Different Types of Water Bodies (*n* = 3)

	Spiked (μM)	Found (μM)	Recovery (%)	RSD (%)
River water	5	4.900	98.00	7.284
	10	9.520	95.20	4.779
	30	29.94	99.80	3.577
Pond water	5	5.046	100.9	5.248
	10	9.992	99.92	3.406
	30	30.56	101.9	2.842
Tap water	5	4.773	95.45	5.915
	10	9.992	99.92	4.682
	30	30.89	103.0	2.571

The validated method was compared with other methods in Table 2. Some methods like LC-MS/MS and SPE-LC-MS methods often involve solid-phase extraction (SPE), derivatization, or complex cleanup steps (e.g., for wastewater or plasma), and GC-FID methods (e.g., SDME) need volatile derivatization and are limited to specific matrices like urine/saliva. Our method is validated for lake water, a matrix with potential interferences (e.g., organic matter). The selectivity of fluorescence can mitigate matrix effects better than UV-based HPLC-PDA, which struggles with overlapping peaks.

**Table 2:** Comparison Table for the Current Approach with Previous Methods

No.	Method	LOQ (μg/L)		LOD (μg/L)	Linear range (μg/L)	Reference
1	LC-MS	0.02		-	0.02–0.50	^30^
2	HPLC (EI and ZMD)	PDA:	500	PDA: 250	500–10000, 100000–1000000	^31^
		TMD:	50000	TMD: 10000		
		ZMD:	50	ZMD: 1		
3	SDME GC-FID	Urine:	1500	450	500–65000	^32^
		Saliva:	1330	0.4	500–65000	
4	UPLC	-		-	0.029–30	^33^
5	LC-MS/MS	Plasma:	1.0	0.75	-	^34^
		Urine:	1.0	2.5	-	
6	LC-MS/MS	Urine:	0.2	-	0.2–400	^35^
7	SPE, LC-MS	Wastewater:	0.02	-	0.020–0.5	^35^
8	LC-MS/MS	Wastewater:	0.0016	0.0048	0.005–1	^20^
9	LC-MS/MS	Wastewater:	0.0019	0.0050	0.005–0.8	^36^
10	Fluorescence	Different water bodies:	82.73	27.58	81–7300	This work

The ratiometric detection of anabasine using CeF_3_ nanocrystals is driven by adsorption-induced surface complexation, as supported by spectroscopic and DLS analyses. Anabasine molecules adsorb onto the CeF_3_ surface via hydrogen bonding between CeF_3_ hydroxyl (−OH) groups and the nitrogen atom in the pyridine ring of anabasine, as evidenced by the disappearance of the O–H stretch (∼3220 cm^–1^) and the emergence of C–H/N–H stretching vibrations in FTIR (Figure 4b). Lewis acid–base interactions between Ce^3+^ centers and the nitrogen of anabasine further stabilize the complex, as confirmed by XPS, which shows subtle binding-energy shifts without new peak formation (Figure S4, Figure 4d, Table S1). DLS measurements reveal a 2-fold increase in hydrodynamic diameter, indicating surface coating and aggregation due to adsorption (Figure 4f). UV–vis studies show minor red shifts and attenuation of anabasine absorption, consistent with these interactions (Figure 4c). Although spectral overlap (Figure 4a) permits an inner filter effect (IFE), the ratiometric fluorescence response indicates specific adsorption and electronic modulation. TRPL lifetime measurements (τ = 8.29 → 8.36 ns) confirm that fluorescence changes arise primarily from static surface interactions rather than dynamic quenching (Figure 4e). Collectively, the dual-emission ratiometric response of the sensor results from the competitive interplay between the CeF_3_ native luminescence and anabasine-mediated emission, highlighting the selectivity and sensitivity of the platform for quantitative anabasine detection, validated through FTIR, UV–vis, XPS, TRPL, and DLS.

**Figure 4:**
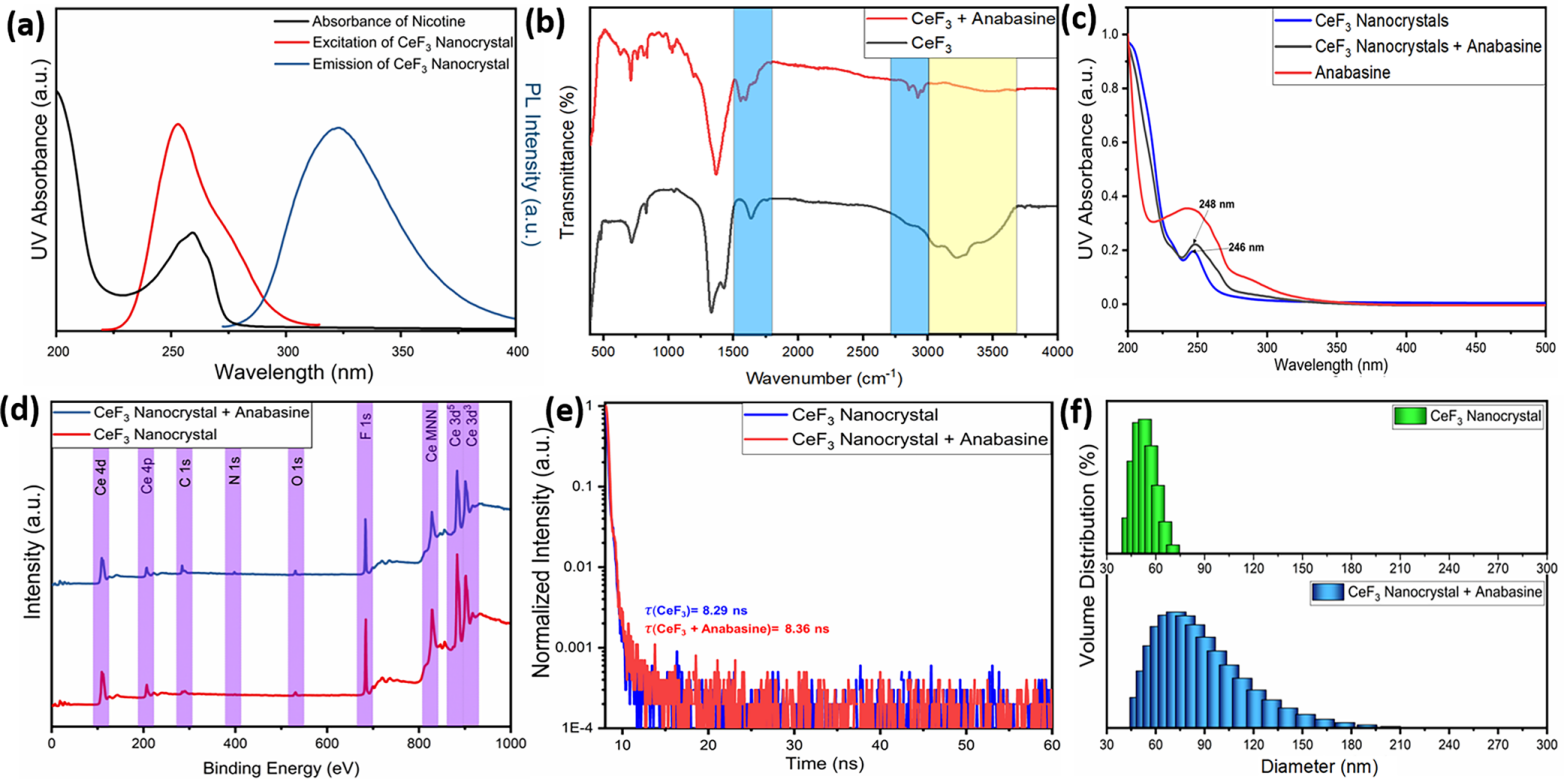
Mechanistic study. (a) Absorbance and excitation/emission graphs, (b) FTIR spectra, (c) UV–vis spectra, (d) XPS studies, (e) time-resolved photoluminescence (TRPL) spectra, and (f) DLS graphs.

## Conclusion

The CeF_3_ nanocrystal-based fluorescent sensor achieves selective anabasine detection via a unique ratiometric response, combining high sensitivity with environmental resilience. Its stability over days, across pH, temperature, and diverse water matrices (lake, river, and tap water), overcomes limitations of chromatographic methods requiring complex preparation. The minimal sample preparation and intrinsic resistance to matrix effects (95–103% recovery) of the probe validate its practicality for field-deployable tobacco pollution monitoring.

This study establishes CeF_3_ nanosensors as practical tools for on-site detection while highlighting the impact of matrix interferences on quantitative fluorescence. Future work may include sample pretreatment or chemometric corrections to enhance robustness in heterogeneous water systems.

## Supplementary Material



## Notes

Experimental details; DLS analysis; photoluminescence response of CeF_3_ nanocrystals toward different analogues and anabasine; anti-interference study; XPS spectra of CeF_3_ nanocrystals before and after anabasine adsorption; and LOD, LOQ, absorption coefficient, and quantum yield calculations (PDF)
