# Supplementary material for: Highly Stable Photoluminescent CeF3 Nanocrystal as a Versatile Probe for Neurotoxic Alkaloid (Anabasine) Sensing via Fluorescence Modulation
Source: Anal Chem. 2026 Feb 9;98(8):5865–71. doi: 10.1021/acs.analchem.5c05171 (PMC12961640; doi:10.1021/acs.analchem.5c05171)
Supplement: Supplementary file 1 [file ac5c05171_si_001.pdf]

Supporting Information

Highly Stable Photoluminescent CeF_3 Nanocrystal as A Versatile Probe for Neurotoxic Alkaloid (Anabasine) Sensing via Fluorescence Modulation

Deepak Dabur[1ǂ], Jie Li[1ǂ], Priyanka Rana[1], Hui-Fen Wu*[1, 2, 3,4,5,6,]

[1]Department of Chemistry, National Sun Yat-Sen University, Kaohsiung, 70, Lien-Hai Road, Kaohsiung, 80424, Taiwan

[2]School of Pharmacy, College of Pharmacy, Kaohsiung Medical University, Kaohsiung, 807, Taiwan

[3]Institute of Medical Science and Technology, National Sun Yat-Sen University, Kaohsiung, 80424, Taiwan

[4]School of Medicine, College of Medicine, National Sun Yat-Sen University, Kaohsiung, 80424, Taiwan

[5]Institute of Precision Medicine, National Sun Yat-Sen University, Kaohsiung, 80424, Taiwan.

[6]Institute of Biopharmaceutical Science, National Sun Yat-Sen University, Kaohsiung, 80424, Taiwan

*Corresponding author: Prof. Hui-Fen Wu (Department of Chemistry, National Sun Yat-Sen University)

Email : hwu@faculty.nsysu.edu.tw

Phone : +886-7-5252000-3955 ; Fax : +886-7-5253909

ǂ Author shares equal contribution.

TABLE OF CONTENTS

Experimental Details……………………………………………………………. S3-S4

Figure S1-S4…………………………………………………………….. S5-S7

Table S1………………………………………………………………….. S8

Calculation (S1-S5) ………………………………………………………… S9-S10

Experimental Details

Materials and Chemicals.

Cerium nitrate hexahydrate ($Ce(NO_3)_3 \cdot 6H_2O$, 99.5%) was purchased from Alfa Aesar, USA. Ammonium fluoride (NH_4F) was purchased from E. Merck KG, Germany. Ammonium hydroxide (NH_4OH, 28%) was purchased from Nippon Shinyaku Co., Ltd., Japan. All the chemicals utilized in experiments were analytical grade without further purification, and the solvent utilized was deionized water (DI water, Merck, Millipore, IQ7000, USA), ethylene glycol (EG, 99.5%) was purchased from Honeywell Riedel-de-Haën, USA.

Characterization and Instrumentation.

High-resolution TEM (HRTEM, JEM-3010, Japan) was utilized to study the structure and morphology of the CeF_3 nanoprobe. The crystalline structure and phase property were studied by X-ray diffraction (XRD, Bruker D8 Advance, Philips, Netherlands). Elemental analysis was performed using high-resolution X-ray photoelectron spectroscopy (XPS, ULVAC-PHI, PHI Quanterall). UV-visible spectrophotometer (Evolution 201, Thermo Scientific, USA) was employed to study the optical properties. Particle size was obtained using a Particle size analyzer (ELSZ-2000ZS, Otsuka Electronics, Taiwan). The fluorescence spectra were studied by a HITACHI F-2700 with a xenon arc lamp at 260 nm excitation.

This study aims to utilize the fluorescent properties of CeF_3 nanocrystals in the detection of anabasine. The sensor was applied to real water samples from three different sources such as river, pond and tap water. All the sample preparation steps and application are provided in the sampling protocols.

Preparation of CeF_3 fluorescent sensing nanoprobe.

First, 0.5 mmol cerium nitrate hexahydrate and 3 mmol ammonium fluoride were dissolved in 2 mL and 1 mL of DI water, respectively. Mix the two chemicals drop by

drop while stirring. Second, add 2 mL of ammonium hydroxide to the mixture and heat at 90°C. After heating and stirring for 1 h, transfer the mixture to a vial and start ultrasonication for 15 min (3s on, 1s off). Third, centrifuge to remove the supernatant, then transfer the solid to a petri dish and dry them in oven at 50 °C. Final, collect the powder and dissolve in ethylene glycol. The viscous solution we get is the CeF_3 fluorescent sensing nanoprobe.

Selectivity and Real sample assay.

The selectivity of the biosensor towards anabasine detection was observed by performing a series of experiments with some nicotine derivatives and biomarkers, such as nicotine, nicotinic acid, nicotinamide, L-arginine, L-asparagine, adenine, and acetaminophen, using the same concentration as that of anabasine at an excitation wavelength of 260 nm.

The river sample was collected from the Erren River, the pond water was collected from Lotus Pond, Kaohsiung, and the tap water was collected from our lab. All the real sample were pretreated by following steps.

First, the samples were put to settle down for 15 min, then were centrifuged for 10 min (6000rpm). Finally, a 0.22 m membrane was used to filter each sample. After that, different concentrations of anabasine were spiked into the as-prepared real samples to get 5, 10, and 30 μM spiked water real samples. After the 30s reaction time with the CeF_3 nanocrystal solution, the measurement of fluorescence intensity was conducted by the fluorescence spectrophotometer at an excitation wavelength of 260 nm.

Figures:

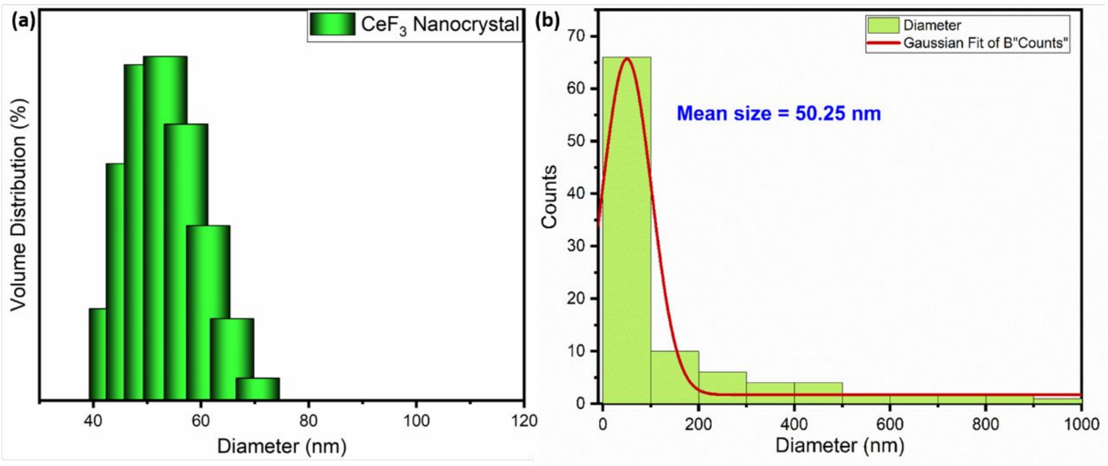

Figure S1. Dynamic Light scattering (DLS) analysis for CeF_3 nanocrystals (a) Volume-based histogram showing the distribution profile of size. (b) Number-based diameter distribution (green bars) fitted with a Gaussian curve (red line).

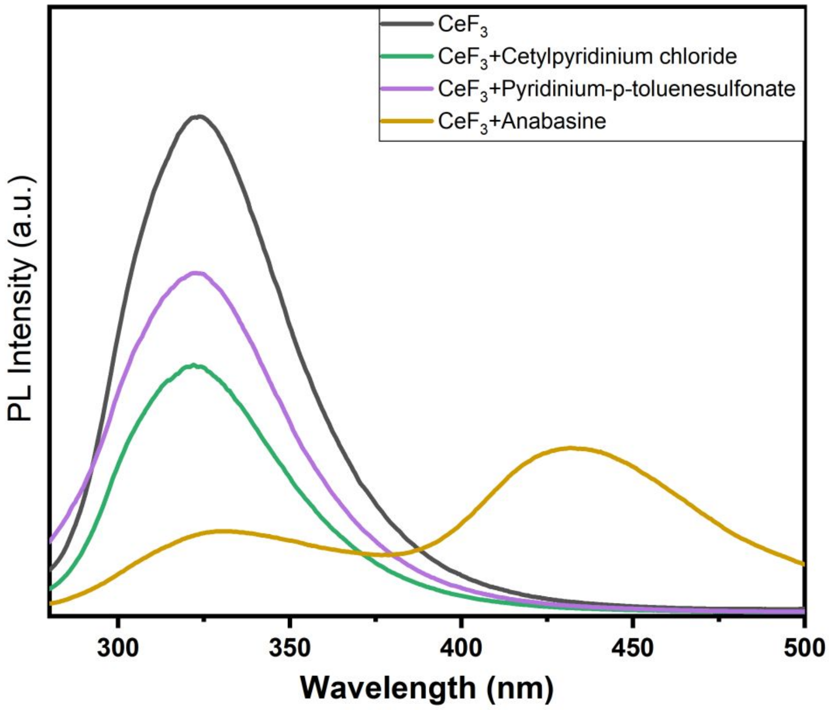

Figure S2. PL response of CeF_3 nanocrystals with nitrogen-blocked pyridine analogues and with anabasine.

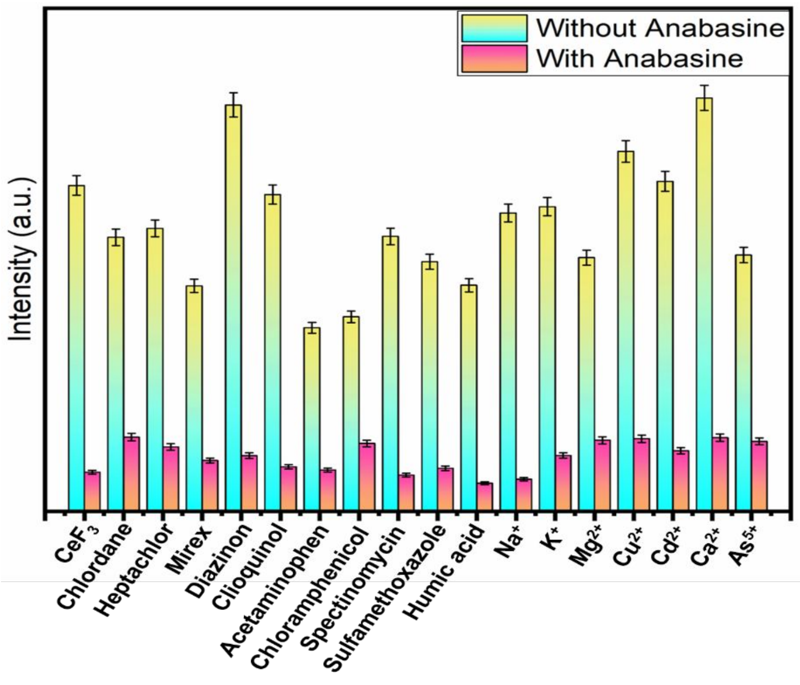

Figure S3. Anti-interference study for anabasine sensing.

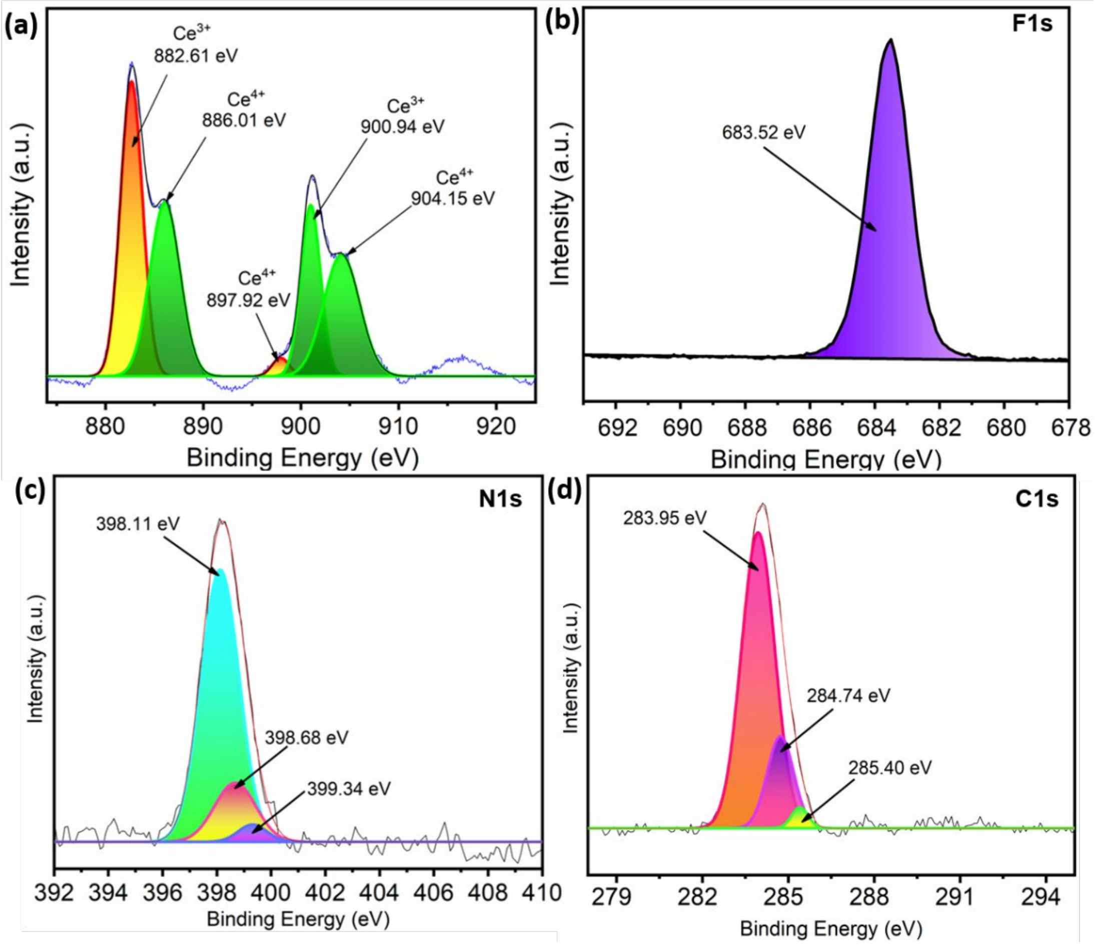

Figure S4. XPS spectra of CeF3 nanocrystals after adding anabasine (a) Ce 3d (b) F 1s (c) N 1s (d) C 1s.

Table S1. Comparison of the results of the high-resolution XPS spectra of CeF_3 pre- and post-adsorption of Anabasine.

Sample	Peak	Binding Energy (eV)	Shift (eV)	Assignment
CeF_3	Ce3d	883.0	-	Ce^{3+}
		886.5	-	Ce^{4+}
		898.1	-	Ce^{4+}
		901.3	-	Ce^{3+}
		904.5	-	Ce^{4+}
	F1s	683.9	-	F
CeF_3 + Anabasine	Ce3d	882.61	0.39	Ce^{3+}
		886.01	0.49	Ce^{4+}
		897.92	0.18	Ce^{4+}
		900.94	0.36	Ce^{3+}
		904.15	0.35	Ce^{4+}
	F1s	683.52	0.38	F
	C1s	283.95	-	C-C/C-H
		284.74	-	C-N
		285.40	-	C=N
	N1s	398.11	-	N-H
		398.68	-	C-N
		399.34	-	C=N

Calculations:

Equations S1 & S2- LOD & LOQ calculation details:

By repeating measure blank sample of CeF_3 nanocrystal for 3 time (n = 3), the sample standard deviation σ will be 0.00048.

$$(\mathbf{S1}) - \text{Limit of Limit (LOD)} = \frac{3.3\sigma}{\text{slope}} = \frac{3.3 \times 0.00048}{0.0095} = 0.17\ \mu\text{M}$$

$$(\mathbf{S2}) - \text{Limit of Quantitation (LOQ)} = \frac{10\sigma}{\text{slope}} = \frac{10 \times 0.00048}{0.0095} = 0.51\ \mu\text{M}$$

Equations S3- Absorption coefficient

Assume that the quantum yield of the synthesis is 100%, we'll get 0.5 mmol CeF_3. Then dissolve 50 mg of it into 25 mL ethylene glycol to get 0.01 M CeF_3 nanocrystal. And we dilute it into 0.0010M

$$\text{Beer's law } A = \varepsilon bc$$

A: the measured absorbance, 0.9043

ε: absorption coefficient ($cm^{-1}M^{-1}$)

b: path length, 1 (cm)

c: concentration, 0.0010 (M)

$$\Rightarrow 0.9043 = \varepsilon \cdot 1 \cdot 0.0010$$

$$(\mathbf{S3}) \Rightarrow \varepsilon = 904.3\ (cm^{-1}M^{-1})$$

By applying above formula and calculations

For CeF_3 with anabasine (0.50 mM), the absorbance is 0.9124

$$\Rightarrow 0.9124 = \varepsilon \cdot 1 \cdot 0.0010$$

$$\Rightarrow \varepsilon = 912.4\ (cm^{-1}M^{-1})$$

Equations S4 & S5- Quantum yield calculation details:

1. The quantum yield of the **synthesized material (CeF_3)** was calculated using the equation as below:

$$\varphi_{CeF_3} = \varphi_{QS} \times \frac{F(AUC)_{CeF_3}}{F(AUC)_{QS}} \times \frac{Absorbance_{QS}}{Absorbance_{CeF_3}} \times \frac{\eta_{CeF_3}}{\eta_{QS}}$$

φ_{QS} : Quantum yield of Quinine sulfate (from reference) = 54.6%

φ_{CeF_3} : Quantum yield of CeF_3

F $(AUC)_{QS}$: Fluorescence Area under the curve of Quinine sulfate = 60,260.0

$F(AUC)_{CeF_3}$: Fluorescence Area under the curve of CeF_3 = 1,01,241

$Absorbance_{QS}$: Absorbance of Quinine sulfate = 0.086

$Absorbance_{CeF_3}$: Absorbance of CeF_3 = 0.196

η_{QS}: Solvent refractive index of the water: 1.333

η_{CeF_3}: Solvent refractive index of the CeF_3: 1.4306

$$(\mathbf{S4})\ \varphi_{\mathrm{CeF_3}} = 54.6\% \times \frac{1{,}01{,}241}{60{,}260.0} \times \frac{0.086}{0.196} \times \frac{1.4306}{1.333} = \mathbf{43.30}\%$$

2. The quantum yield of the **synthesized material after anabasine** addition was calculated using the equation as below:

$$\varphi_{\mathrm{Ce}F_3+Anabasine} = \varphi_{\mathrm{QS}} \times \frac{\mathrm{F}_{\mathrm{Ce}F_3+Anabasine}}{\mathrm{F(AUC)}_{\mathrm{QS}}} \times \frac{\mathrm{Absorbance_{QS}}}{\mathrm{Absorbance}_{\mathrm{Ce}F_3+Anabasine}} \times \frac{\eta_{\mathrm{Ce}F_3+Anabasine}}{\eta_{\mathrm{QS}}}$$

φ_{QS} : Quantum yield of Quinine sulfate (from reference) = 54.6%

$\varphi_{\mathrm{Ce}F_3+Anabasine}$: Quantum yield of CeF_3

F $(AUC)_{QS}$: Fluorescence Area under the curve of Quinine sulfate, 60,260.0

$F(AUC)_{\mathrm{Ce}F_3+Anabasine}$: Fluorescence Area under the curve of CeF_3+Anabasine, 31,539.0

$Absorbance_{QS}$: Absorbance of Quinine sulfate, 0.086

$Absorbance_{\mathrm{Ce}F_3+Anabasine}$: Absorbance of CeF_3+Anabasine, 0.22

η_{QS}: Solvent refractive index of the water: 1.333

$\eta_{\mathrm{Ce}F_3+Anabasine}$: Solvent refractive index of the CeF_3: 1.4306

$$(\mathbf{S5})\ \varphi_{\mathrm{CeF_3}} = 54.6\% \times \frac{31{,}539.0}{60{,}260.0} \times \frac{0.086}{0.220} \times \frac{1.4306}{1.333} = \mathbf{12.0}\%$$
